# Robustness in experimental design: A study on the reliability of selection approaches

**DOI:** 10.5936/csbj.201305002

**Published:** 2013-06-30

**Authors:** Stefan Brandmaier, Igor V Tetko

**Affiliations:** a Helmholtz Zentrum München - German Research Center for Environmental Health (GmbH), Institute of Structural Biology, Neuherberg D-85764, Germany; b Chemistry Department, Faculty of Science, King Abdulaziz University, P. O. Box 80203, Jeddah 21589, Saudi Arabia; c eADMET GmbH, Ingolstaedter Landstrasse 1, Neuherberg D-85764, Germany

**Keywords:** Design of experiments, outliers, compound selection, representative sampling, similarity selection, descriptor selection

## Abstract

The quality criteria for experimental design approaches in chemoinformatics are numerous. Not only the error performance of a model resulting from the selected compounds is of importance, but also reliability, consistency, stability and robustness against small variations in the dataset or structurally diverse compounds. We developed a new stepwise, adaptive approach, DescRep, combining an iteratively refined descriptor selection with a sampling based on the putatively most representative compounds. A comparison of the proposed strategy was based on statistical performance of models derived from such a selection to those derived by other popular and frequently used approaches, such as the Kennard-Stone algorithm or the most descriptive compound selection. We used three datasets to carry out a statistical evaluation of the performance, reliability and robustness of the resulting models. Our results indicate that stepwise and adaptive approaches have a better adaptability to changes within a dataset and that this adaptability results in a better error performance and stability of the resulting models.

## 1. Introduction

Experimental design techniques are crucial in terms of time and cost efficiency as well as to minimize the number of animal experiments. Reliable testing strategies are essential, especially in the course of the REACH legislation,[1] which includes the requirement that every chemical compound produced in/or imported into the European Union in an amount of more than one ton, has to be registered regarding a number of endpoints. But the application of selecting a representative and descriptive sub-sample from the chemical space of interest, and using it for the calculation of prediction models, is not only limited to risk assessment within REACH.[2, 3, 4] Also tasks as large scale scanning of chemical databases,[5] QSAR modeling,[6] drug target evaluation[7] or other pharmaceutical applications require systematic approaches to select representative subsamples.

The variety of concepts to address these problems in computational chemistry and QSAR modeling is widely spread,[8, 9] but most of them can be reduced to one of three basic ideas. Firstly, the selection of compounds with maximum dissimilarity, which is based on the theory that the most distinct compounds contain the most diverse information. This idea/theory is optimal for linear modeling. The D-Optimal criterion[10, 11, 12] and the Kennard-Stone algorithm[13] belong to this group of approaches. Secondly, the similarity selection aims to find compounds with high representativeness for the whole collection of relevant compounds. Approaches referring to this concept, e.g. the most descriptive compound selection (MDC),[14] usually select compounds from densely populated regions of the chemical space. Thirdly and lastly is an approach that aims to cover the whole chemical space of interest. The full factorial design[15] and space filling designs[16] are examples thereof. Recently, approaches that utilized hierarchical or density based clustering techniques were proposed.[9, 17] In our last study[18] we presented the advantages of an adaptive approach that combines a dissimilarity selection with an iteratively refined representation of the chemical space, by taking into consideration the information about the analyzed property that accumulates in the experimental process.

In QSAR modeling and chemoinformatics the focus within the evaluation of a novel approach is often exemplified on a particular dataset. Statistical evaluations, taking performance measures such as reliability and robustness of an approach into consideration are rare.[19] Due to chance correlations, this can result in misleading conclusions about the applicability of an approach. Furthermore stability, which is the ability of adapting small changes in a dataset, or to process structural outliers in a data collection, also needs to be taken into consideration. This is a quality criterion, which is as important as the performance itself.

In this study we present DescRep, a stepwise adaptive approach combining an iteratively refined descriptor selection with a sampling based on the concept of representative compounds. We compare this approach to experimental design strategies, which are commonly used in chemistry. An evaluation pipeline was implemented and applied to an ensemble of randomly selected subsets of three datasets, each with an endpoint relevant for REACH. We show that in comparison to the traditional approaches that select all compounds at the same time, DescRep performs significantly better.

We exemplify the importance of a statistical evaluation by investigating the effects of small changes in the underlying dataset on both the composition of the selected compounds and the performance of the resulting model. Furthermore, the collected datasets are extended with concerted structural outliers, to evaluate their influence on the selection approaches and the resulting models. Our results indicate that stepwise approaches, DescRep in particular, contribute to stability and reliability in experimental design.

We investigate the benefits of a representation of the chemical space, which takes the correlation to the target property into consideration, and consequently arranges the compounds to a certain reference endpoint. Finally, we analyze our results with respect to the variability and adaptability of the examined approaches.

## 2. Materials and Methods

### 2.1. Materials

To compare and evaluate the selection approaches, we collected three datasets, which vary in several criteria. The respective endpoints of these datasets, which were also used in our previous study,[18] were a physicochemical property, boiling point, a soil sorption coefficient, logK_OC_, and environmental aquatic toxicity against freshwater fish fathead minnow.

We extracted a collection of boiling point values from the Estimation Programs Interface (EPI) suite data.[20] The compounds within the dataset were restricted to halogenated compounds, containing bromine, chlorine and/or fluorine. As no further structural filters were applied, this set still provided a broad diversity regarding molecule size and chemical structures. We did not apply any kind of structural filter to the other datasets. The second dataset was based on the collection of logK_OC_ values by Meylan et al.[21] logK_OC_ is the log scale of the adsorption coefficient of a contaminant in the organic fraction of the soil. The endpoint for the toxicity dataset was the log scaled aquatic LC_50_ value on the fathead minnow. The measurements were taken from the fathead minnow acute toxicity database[22] of the Environment Protection Agency (EPA).

All datasets were free of duplicate compounds. Measurements providing intervals of minimum or maximum values were excluded. In order to avoid problems in descriptor calculation, inorganic compounds, radicals, charged molecules and salts were filtered out. The final dataset for the boiling point contained 1198 compounds, the datasets for logK_OC_ and for toxicity on the fathead minnow contained 648 and 535 chemicals, respectively.

For each dataset, a collection of two types of descriptors was calculated. The first type was calculated using the ALOGPS 2.1 program[23] and contained two descriptors: solubility and lipophilicity of molecules. ALOGPS was the top-ranked model for prediction of logP.[24] The second type included E-State indices.[25, 26] These are electrotopological descriptors calculated for each atom and each bond in a compound and then summed according to their types over all atoms. The number of descriptors for the second type is determined by number of different chemical groups and thus it was not a fixed one. On our datasets, we calculated 179, 220 and 230 descriptors for logLC_50,_ logK_OC_ and the boiling point dataset, respectively. The Online CHEmical database and Modeling environment (OCHEM)[27] was used for the calculation of the descriptors. To represent the chemical space of each dataset the descriptors were normalized to a [0,1] range. The rationale to use normalization instead of standardization is that standardization works on the underlying assumption that the objects are normally distributed. This assumption is not true for descriptors determined for chemical groups, e.g., in particular for the E-State indices. As they are linked to the presence of certain substructures, for most compounds, their value is just zero.

One of the aims of this study was to investigate the influence of structurally diverse compounds on the selection and accuracy of the resulting models. Therefore each of the three datasets was extended by the inclusion of a compound, which was characterized as a structural disrupter. We defined a structural disrupter as a data point that (a) influences the recalculated loadings of the first or the second principal component in such a manner that the principal properties represented by these components are changed and (b) results in one or more instances in the data set that are – according to the distribution of the instances in that principal component – at least five standard deviations from 97% of all other compounds.

Structural outliers like the ones used in this study are not artificial, but can result from several reasons, e.g. (a) from few compounds within the dataset, which have a specific chemical group that is different from other compounds and functionally is not relevant, (b) from the choice of a specific descriptor set, or (c) from a certain procedure within the multivariate analysis (centering or not the data, usage of raw, normalized or standardized data).

**Figure 1:**
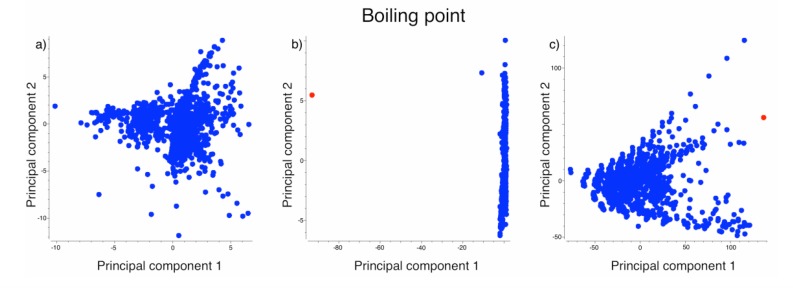
The change in the principal components view due to one structural outlier in the dataset. The principal components were calculated for the dataset with (b, c) and without (a) structural outlier. ALOGPS and E-State indices were used (a, b), as well as DRAGON descriptors (c). The protocol to calculate the principal components was always the same.

The structural outliers in our study were (a) ethyl 2-chloro-3-[2-chloro-5-[4-(difluoromethyl)-3-methyl-5-oxo-1,2,4-triazol-1-yl]-4-fluorophenyl]propanoate (carfentrazone-ethyl) for the boiling point dataset, (b) (1R,4aR,4bS,7S,10aR)-7-ethenyl-1,4a,7-trimethyl-3,4,4b,5,6,8,10,10a-octahydro-2H-phenanthrene-1-carboxylic acid (isopimaric acid) for the logLC_50_ dataset and (c) (1,2-dimethyl-3,5-diphenyl-pyrazol-1-yl) methyl sulfate for the logK_OC_ dataset. All these three compounds were retrieved from the same source as the rest of the respective dataset. Fig. 1a) shows the first two principal components of the boiling point dataset without outliers whereas Fig. 1b) shows the first principal components of the same dataset with the structural disruptor. The structural disrupter has a red color. The principal components were derived from the whole set of normalized ALOGPS descriptors and E-State indices and thus no variable selection was performed. Furthermore, the data were not centered before the orthogonal transformation.

**Figure 2:**
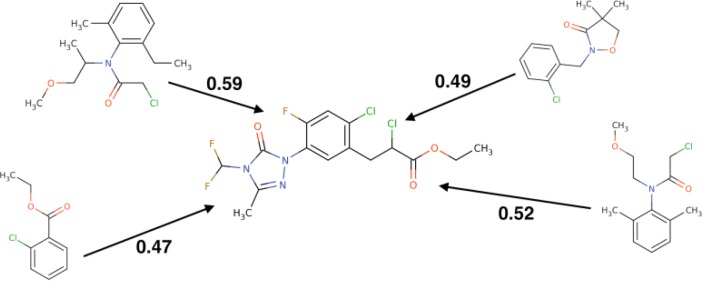
The structural outlier and similar compounds in the dataset.

To show how the concerted outlier for boiling point structurally fits into the dataset, we calculated its Tanimoto distance to all other compounds. ISIDA fragments[28] were used therefore. Fig. 2 shows the outlier in the center and the four most similar compounds around. The value assigned to the edges indicates the similarity score. It is obvious that the outlier is a larger molecule and contains a triazole group, which is absent in other compounds. Such types of outliers could naturally happen to be present in the datasets. The appearance of such outliers depends on the used descriptors. Fig. 1c shows, if Dragon descriptors[29] are used, this compound is not anymore an outlier (although it is located at the periphery of the data cloud). Indeed, Dragon software calculates many more descriptors and in their space the analyzed molecule does not have descriptors, which make it to be the outlying point in the PCA space. Thus, a property of a molecule to be a structural outlier depends on the used set of descriptors, i.e. on the representation of the molecule.

### 2.2. Methods

#### 2.2.1. Static experimental design approaches

We implemented several commonly used experimental design strategies to evaluate and compare the robustness and reliability of selections derived by these approaches. All of the following approaches were applied to five principal components derived from a principal component analysis of the descriptor space. The results of our recent study[18] showed that a search space of that dimensionality worked equally well for all datasets.

##### D-Optimal design

The D-Optimal design[10, 11, 30] uses the determinant of the information matrix to evaluate all possible subsets of *n* out of *m* compounds (*n* < *m*). The set with the maximum entropy,[31] and therefore the most distinct one, is the one with the maximum value on the determinant. We used the D-Optimal criterion in combination with the Fedorov heuristic[32] to minimize the runtime requirements. The D-Optimal design works well for linear models, but reveals a bias towards outlier selection in higher order spaces.[8

##### Kennard-Stone algorithm

Starting from an initially selected compound, the Kennard-Stone algorithm[13] selects compounds in a fixed order. In this study, the initial selected compound was the central point within the dataset, which was defined to be the compound with the minimum sum of distances to all other compounds. From this initial seed, each step in the selection extends the chosen compounds by that one that has the highest Euclidean distance to its closest neighbor within the previously selected ones. The disadvantages of this approach are similar to those of the D-Optimal design.

##### Space filling design

The space filling design as a variant of the full factorial design is working by partitioning the chemical space into subspaces. These subspaces are derived by dividing each axis into the same number of bins. Therefore, for a number of *b* bins and a number of *a* axes, the number of resulting subspaces is *b*^*a*^. From each of these resulting subspaces a compound is selected as representative, but as the compounds are not equally distributed in the chemical space, subspaces can be completely without a representative compound. It is therefore difficult to fix the number of finally selected compounds. In our implementation, the number of bins each axis is divided into is not prefixed, but automatically detected to be optimal for the desired number of compounds to be selected as described elsewhere.[33] The compound selected as representative for each subspace is the one with the lowest Euclidean distance to the center of the subspace. Since the number of subspaces is exponentially increasing with each dimension in the search space, we fixed this approach to work on three principal components.

##### Most descriptive compound selection (MDC)

The most descriptive compound selection is working in a sequential manner. Initially all compounds get assigned a score displaying their representativeness for all other compounds. In each step the compound with the highest score is selected and all other scores are updated by eliminating the deduced information content of the selected compound. Our implementation of this approach was based on the work of Hudson et al.[14] Instead of using the suggested stop criterion, we selected a fixed number of compounds.

##### Random selection

Additionally, to provide a reference a random selection was used for comparison.

#### 2.2.2. Adaptive experimental design approaches

The adaptive experimental design approaches we use in this study, work in a stepwise manner, where each step consists of two phases. In the first phase the representation of the chemical space is refined. This is done by using the preliminary gathered information from the target property and analyzing its correlation to the chemical space. In the second phase a selection algorithm is executed on the newly arranged chemical space. The selection is hereby taking all previously selected compounds into consideration. These phases are executed in an alternating way until a prefixed number of compounds are reached.

The idea behind the rearrangement of the chemical space is to adjust the design of an experiment to a certain endpoint and consequently to reach a faster increase in the resulting model performance. Experimental designs derived from PCA space are not aligned to the target property, but are identical for the same selection algorithm and executed on the same compound collection, regardless of the endpoint. For this reason they are unspecific and most probably not optimal.

##### PLS-Optimal

PLS-Optimal is an adaptive approach that combines the D-Optimal criterion with the partial least squares technique (PLS). The representation of the chemical space within this approach is realized with PLS components instead of PCA components. The principal components derived by a PCA are ranked by their variance in descriptor space; in contrast the PLS latent variables are ranked by their correlation to the target property. The latent variables in our implementation are derived from a PLS model on the pre-selected compounds. In our previous study on this approach, we showed by taking the correlation to the target property into consideration, we could significantly improve the performance of the D-Optimal selection criterion.[18]

##### DescRep

The new approach, DescRep works in a similar way as PLS-Optimal. However, it combines a similarity-based approach (instead of a dissimilarity based one) with a representation of the chemical space using selected descriptors (instead of PLS components). As for PLS-Optimal, the preselected compounds are used as reference information to evaluate the most important descriptors.

*Descriptor selection*: The search space for DescRep is spanned by a fixed number of selected descriptors. The selection process follows a simple idea and is therefore straight and efficient.

In the first step a scoring list *S*, containing the correlation coefficient of each descriptor to the target property, is built. This correlation coefficient is derived only from the preselected compounds, which are already ‘measured’. Additionally, the correlation matrix *M*, containing the absolute pairwise correlation of any combination of two descriptors, is built.

According to the scoring list, the descriptor that should be selected is the one with the highest score, which is initially equivalent to the one with the highest correlation to the target property. After the selection of a descriptor *x* the scores are updated to avoid pairwise correlations in the final selection. Regarding the compound *i*, its score *S*_*i*_ gets updated to *S*_*i*_ * (1 - *M*_*ix*_)^3^. Thereby the scores of descriptors, which are highly correlated to the preselected ones are decreased, which helps to avoid the selection of redundant information. The underlying idea of selecting variables with a high correlation to the target property and the elimination of inter-correlated variables is similar to partial least squares.[34] This procedure is repeated until a predefined number of descriptors are selected.

*Selection of compounds*: As a stepwise experimental design procedure requires a selection method, which is able to take a preselected initial seed of compounds into consideration, it is not possible without further ado to use the MDC selection, as its concept of ranking distances cannot be adapted to this precondition. Therefore we developed a selection method based on the idea that structural similarity of compounds also conditions similar values regarding a certain endpoint.

We select an initial seed of compounds starting from a k-Means-based partition of the chemical space (represented by the principal components) into a predefined number of clusters. The initial seed contains the most representative compound of each cluster. The most representative one is hereby defined as that compound with the lowest sum of pairwise distances to all other compounds within the same cell. The k-Means clustering was initialized 15 times with randomly assigned starting compounds. The finally picked clustering was the one with the lowest sum of pairwise differences within each cluster.

In each further step the chemical space is represented by a selection of descriptors based on the preselected compounds. The preselected compounds are extended by new ones, which are assigned to be the most informative ones for all other compounds based on a priority score (*PS*) calculated for each compound. *PS* estimates how well a compound is represented by all previously selected compounds.

Initially, all compounds are assigned a *PS* of 1.0 and the distance matrix *DM*, containing the pairwise distances between all compounds, is calculated. The distance matrix (normalized to [0,1] range) is used as *PS* to select the first compound. The use of such matrices to represent datasets has been shown useful in numerous publications.[35, 36

For all following compounds, the normalized pairwise distances of *N* preselected compounds to the remaining compounds in the dataset are used to determine how well each compound is already represented and select the least represented ones. Each compound *x* within the set gets assigned a correction factor *CF*_*xi*_ for each preselected compound *i*. The correction factor refers to a hyperbolic distance function and it is used to adjust the *PS* to the preselected compounds.

$$PS_{x\_\mathrm{new}}=PS_{x}*\prod_{i=1}^{N}CF_{\mathrm{xi}}$$

The correction factor is calculated as

$$CF_{\mathrm{xi}}=\left(1-{\left(1-DM_{\mathrm{xi}}\right)}^{\exp}\right)$$

The exponent *exp* is not fixed, but depends on the distribution of the data in the descriptor space and the number of compounds to be selected. It is recalculated in each selection cycle. Referring to the most central point within the dataset (which is again defined as the one with the lowest sum of pairwise distances to all other compounds) *exp* is determined as the value for which the number of preselected compounds and compounds to be selected in the present cycle has to have a value of (1-*DM*_*xi*_)^*exp*^, which is higher than a given threshold. This procedure is a variation of the MDC,[14] which is based on distances, instead of reciprocal ranks. The threshold value we used in this study was lamda = 0.75. For the calibration datasets on density, bioconcentration, lipophilicity and solubility were used. We experimentally determined that this is an appropriate value. This additional feature of the recalculated exponent enables one to also handle exceptional data distributions. The method is not sensitive to the parameterization. We tried different versions of lamda within the range of 0.5-0.9 and did not observe significant changes in the method performance. Moreover, it should be mentioned that parameterization and an appropriate distance function are general issues for similarity-based selections.[14

Based on these prior conditions, the correction factors for all combinations of not yet selected compounds are calculated. The collection of compounds finally selected for testing is the one that minimize the sum of priority scores over all compounds.

#### 2.2.3. Validation

All three datasets were split into two partitions. The first partition (design set), containing 84% of the compounds, was used to execute the selection approaches and the second partition, containing the remaining 16% of the compounds, was used as a respective validation set. A split of that size was chosen, as it guarantees that two randomly generated design sets have 68% (approximately two third) of compounds of the whole dataset in common. To retrieve a statistically meaningful foundation to evaluate and compare the approaches, 250 of these splits were generated. Therefore each compound is present in average in 210 of the design sets and in 40 of the validation sets. Each of the splits was used for the evaluation of each of the selection approaches. For all datasets, we used the approaches to select samples containing 5, 7, 10, 15, 20, 25, 30, and 40 compounds. For the boiling point dataset, additional samples containing 50 and 60 compounds were selected. The selection process for the static approaches was started from scratch for each sample size, whereas for the adaptive approaches the selection process was strictly based on the sequence as mentioned above. Thereby the compounds selected in each previous step are in the next step used as a known seed and the newly selected compounds just extend this seed.

The evaluation of each selection is obtained by building a PLS regression model. The number of latent variables to be used for the final model was determined in a five-fold cross validation on all selected training set compounds using the coefficient of determination as criterion for the optimal number.[34

The models were built using all normalized descriptors and not by using only the principal components that spanned the search space. These models were applied to the validation set which contained all compounds that have been excluded from the selection process. Therefore the models performance on this dataset provides an independent measurement of the prediction quality on new compounds. The criteria for the model evaluation were the root mean squared error (RMSE) and the correlation coefficient between observed and predicted values. We estimate the significance of the difference in performance according to a binomial test (the binomial distribution with *N*=250 trials). All mentioned significant differences in the article had *p* < 0.05.

## 3. Results and discussion

### 3.1. Model performance

**Figure 3:**
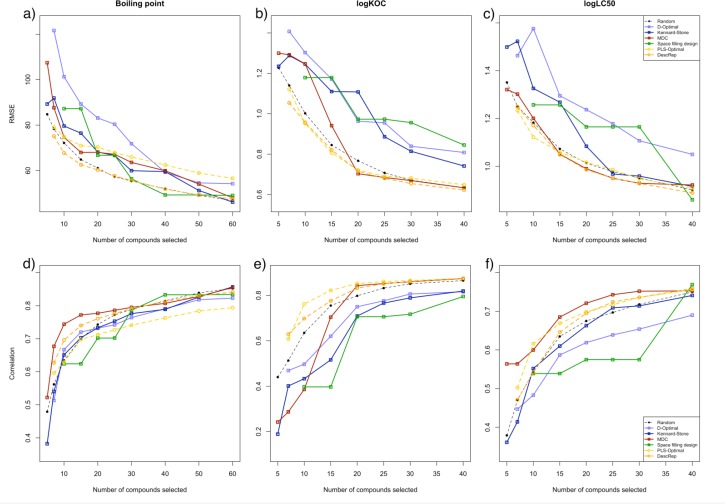
Average performance of the models resulting from the selections of the examined approaches, displayed as a-c) RMSE and d-f) correlation coefficient on the datasets for a, d) boiling point (°C), b, e) logKOC and c, f) logLC50. The stepwise approaches are displayed by the dashed orange lines (DescRep) and the dashed yellow lines (PLS-Optimal). The color assigned to the random selection is black, red for the MDC selection, green for the space filling design and blue for the dissimilarity selections.

To enable a comparison of the quality of the models resulting from the examined selection approaches, we calculated the average RMSE performance and the average correlation coefficient for each number of compounds selected. Fig. 3a–c) shows the results of this comparison using the prediction error, whereas Fig. 3d–f) shows the comparison using the correlation. The x-axis displays the number of selected compounds and the y-axis the measurement of quality.

The first general observation on all of the datasets and selection approaches is that with an increasing number of selected compounds the average error decreases, whilst the average correlation in the models increases. This is expected as a larger number of molecules provide an increase in the amount of information obtained and thereby enables one to build a better model. Furthermore, for all datasets the stepwise approaches reach a good performance, which is constantly within the range of the best approaches. PLS-Optimal reveals problems with the BP dataset, these problems were explained in our previous study[18] with the similarity between the loadings of the PLS latent variables and the loadings of the principal components. The average performance of models derived from compounds selected with DescRep is also the best for the boiling point.

A further observation is the smooth hyperbolic development of the average error performance on the 250 splits for each dataset. Whereas the static approaches result in unexpected deviations, there are no irregularities for the stepwise approaches, neither in the error, nor in the correlation development. MDC is the only systematic approach that derives selections resulting in a performance, which is as comparably good, although it reveals similar problems as the other approaches for the boiling point and the logK_OC_ dataset until 20 selected compounds.

The models derived from the selection of both stepwise approaches show a low initial prediction error. The performance of PLS-Optimal for seven selected compounds is better than e.g. than that of the D-Optimal criterion for 25 selected compounds on the boiling point dataset, for 15 compounds on the logK_OC_ dataset and on the logLC_50_ dataset for 20 compounds. Further worth mentioning, is the good performance of models resulting from the random selection. Like the stepwise approaches, the random selection provides models that reliably decrease in average error and increase in average correlation for a growing number of compounds selected.

Regarding the correlation coefficient, MDC shows the fastest increase of all examined methods for the boiling point and the logLC_50_ dataset. The models from the MDC selection on the logK_OC_ dataset, clearly show a worse initial correlation for less than 20 selected compounds. Although the convergence in the correlation for the stepwise approaches is not that fast, it works equally well on all datasets and it is still faster in comparison to all other systematic approaches.

Referring to the binomial test, we found that the observed improvements in the resulting models derived with DescRep are of high statistical significance (p < 0.001) for the range of 7 to 20 selected compounds for the boiling point dataset, 7 to 25 selected compounds for logK_OC_ and 15 to 40 selected compounds for logLC_50_, when compared to the random selection. Regarding a comparison of PLS-Optimal with a random approach, we observed this level of statistical significance for the range of 10 to 25 selected compounds for the logK_OC_ dataset and 7 to 15 selected compounds for the logLC_50_ dataset. Furthermore, DescRep performed better than MDC (the best static approach) with high statistical significance (p < 0.001) over the whole examined range for the boiling point and for 5 to 15 selected compounds for the logK_OC_ dataset.

### 3.2. Consistency and stability

**Figure 4:**
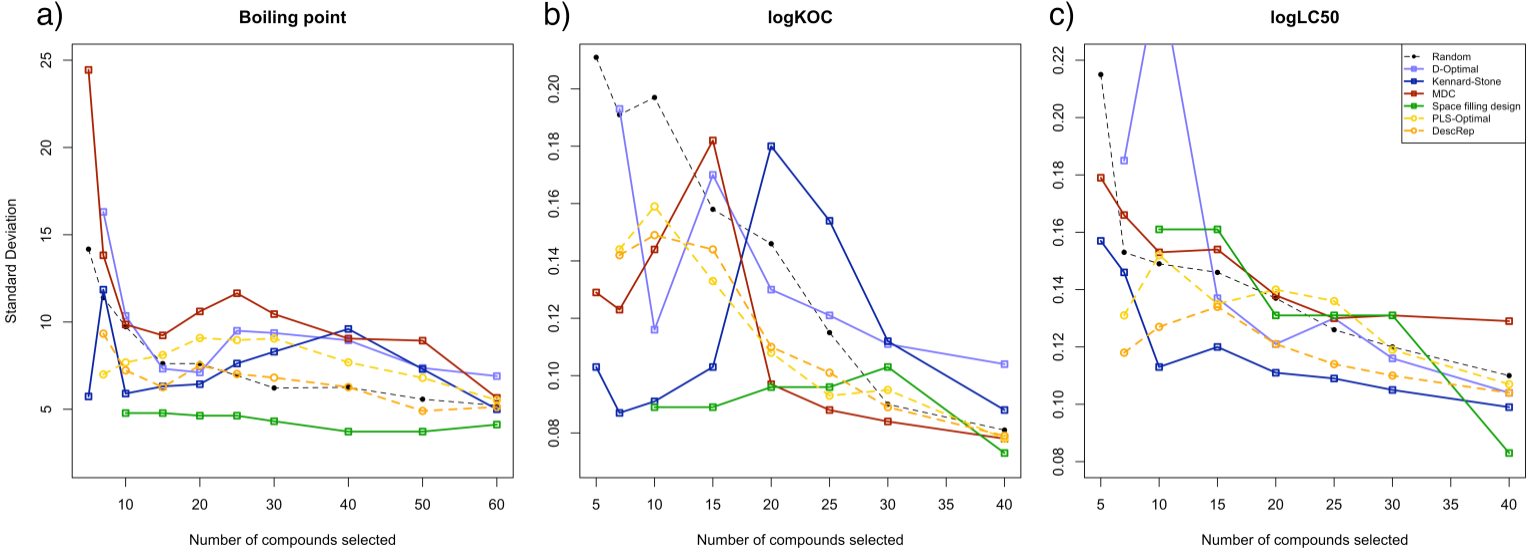
Comparison of the standard deviation of the selection approaches on a) the boiling point, b) the logK_OC_ and c) the logLC_50_ dataset.

In addition to the average error, the reliability and stability in the performance of the resulting models have to be taken into consideration. We therefore calculated the standard deviation within the models of the 250 trials on each dataset, for each number of selected compounds, and for each selection approach. The results are shown in Fig. 4. The colors are identical to that of Fig. 3 and the y-axis displays the standard deviation, whereas the x-axis displays the number of compounds selected.

The first general observation is that with an increasing average error the standard deviation also increases for most of the approaches. The exceptions are the models derived with the Kennard-Stone algorithm on the logK_OC_ dataset, as they show an increase in standard deviation by a factor of two for 20 compounds selected in comparison to 10 compounds selected. Regarding the random approach, the variations in the initial performance are high. This high level of uncertainty in the resulting models is why this approach is frequently found inappropriate, in spite of its reasonable average performance.

The space filling design has the lowest standard deviation for the resulting boiling point and logK_OC_ models, whereas the MDC approach, the only systematic method that could at least partially reach the same performance as the stepwise approaches, has a significantly higher standard deviation than DescRep on all datasets and for the whole range of selected compounds.

**Figure 5:**
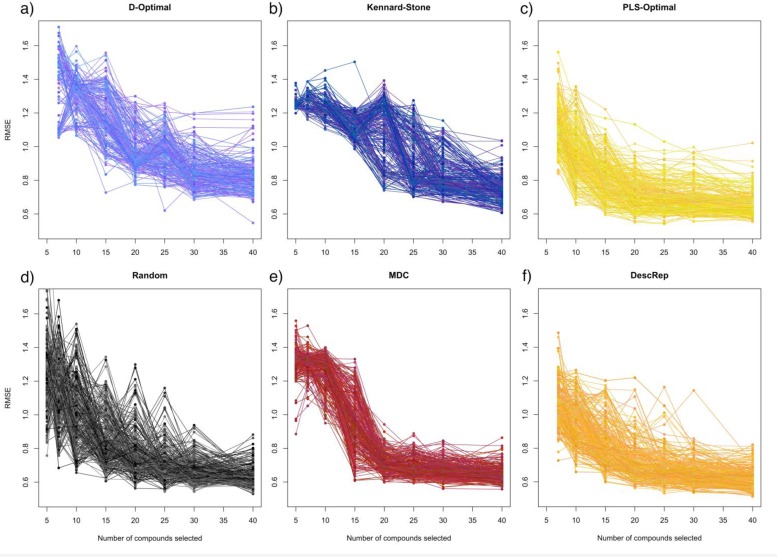
All 250 models on the logK_OC_ dataset.

Fig. 5 provides a more detailed insight into the distribution of performance of the resulting models and the development of particular validation splits. It shows the RMSE development of all 250 validation splits on the logK_OC_ dataset for a) the D-Optimal criterion, b) the Kennard-Stone algorithm, c) PLS-Optimal, d) the random selection, e) the MDC selection and f) DescRep.

Both stepwise approaches produce only a small number of low performance outliers, whereas the majority of the validation splits results in models with quite similar performance. Additionally, for almost all splits, the initial performance of the resulting model is lower than for the other approaches and the error performance shows a fast convergence. Furthermore, the error on the validation splits steadily decreases for a higher number of selected compounds. Especially for the dissimilarity approaches this is not the case, e.g. Kennard Stone selection delivers a worse model for 20 than for 15 selected compounds. And for the D-Optimal criterion these deviations of worse models for a larger training set are widely spread over the whole range of selected compounds.

### 3.3. Outlier robustness

**Figure 6:**
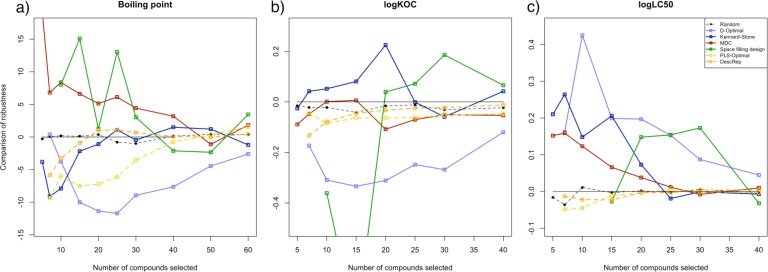
Effects of the structural outliers to the selection approaches to the examined datasets, displayed by the difference in average RMSE performance.

All calculations were repeated with the extended sets, each containing a structural outlier. To compare the effects of such outliers to models derived by the selection approaches, we determined the difference in the average RMSE between the sets without and the sets with outliers. The results are shown in Fig. 6. The colors are in accordance with all previous figures, and the y-axis displays the difference in average performance. Approaches that result in models with a better performance on datasets with structural outliers, have positive values, those performing better on sets without structural outliers, have negative values.

Both stepwise approaches show only small deviations in the resulting models. Apart from an initial better performance of PLS-Optimal on the boiling point dataset without structural outlier, the selections derived with the adaptive approaches perform equally well on the extended datasets. Also the MDC selection is mostly resistant to the outlier, whereupon a tendency to deliver better selections on datasets with outliers is observable. Contrary, the effect of only one additional compound on the other approaches was incalculably. The models derived with the space filling design, the D-Optimal criterion on principal components and the Kennard-Stone algorithm, have no clear tendency towards the original or the modified dataset. The sign of the difference in the average error of the resulting models differs from dataset to dataset. This is also the case for the space filling design, even within the logK_OC_ dataset.

## 4. Discussion

Both stepwise approaches: DescRep and PLS-Optimal, performed equally well on the analyzed datasets. The error performance of their resulting models is in general lower than that of the approaches that select all compounds at the same time. The development of the error is smooth and reliable. Both methods reveal a lower standard deviation compared to MDC, which is the best performing non-stepwise approach. The average correlation coefficient develops in a similar way. Neither on the logLC_50_ dataset, nor on the logK_OC_ dataset any of the classic approaches was performing better than the stepwise approaches and on the boiling point dataset, none of the classic approaches performed better than DescRep.

This good performance can also be observed in the depiction of the specific models in Fig. 4. At large, for both stepwise approaches an increase in the number of selected compounds results in a decrease of the error. This is not the case for the Kennard-Stone algorithm and the D-Optimal criterion where high variations in performances were observed.

Overall, DescRep is superior over the PLS-Optimal approach, as it was able to deliver high quality performance models even on the dataset where the performance of PLS-Optimal was not ideal. Nevertheless, the decrease in the performance accuracy of PLS-Optimal on the boiling point can be easily explained and is therefore avoidable. The boiling point dataset resulted from a correlation between the PLS components and PCA components.[18] It is important that DescRep is not affected with such problems.

**Figure 7:**
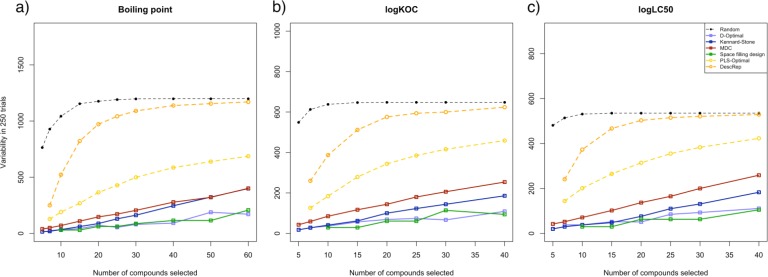
Variability in selection.

To investigate the major difference between the stepwise and the non-stepwise (static) approaches, we analyzed the compounds selected by the different methods and compared their distribution in the design sets. To compare the variability in the selections of the methods, we counted the number of different compounds selected in the 250 trials. We found a significant difference between the stepwise and the static approaches. Whereas the systematic approaches, which select all compounds at the same time, have a comparably small pool of compounds that are selected, the stepwise approaches are resulting in a higher variety of selected compounds. This variability is shown in Fig. 7. The stepwise approaches have a better adaptability to small variations in the datasets. The observance that PLS-Optimal has a lower variability in selection than DescRep is coherent as the D-Optimal criterion also has lower variability than MDC. Still, the variability of DescRep is significantly lower than that of the random approach. This shows that the selection process is still systematic and contributes to better performance of DescRep compared to random selection.

It is interesting to note that despite step-wise approaches have a higher variation in the number of selected compounds, the models developed with these compounds have lower variation compared to those developed using static approaches. The contradiction clearly indicates that the variability in selected compounds in both stepwise approaches is a meaningful adaption to changes in the dataset. Whilst the variation within the selected compounds is clearly increased for the MDC approach compared to the stepwise approaches, the resulting models show a significantly higher standard deviation than the stepwise approaches.

Additionally, not only referring to the adaption of small variations in the dataset, but also in terms of outlier adaption, the stepwise approaches show a convincing performance. The average error of the resulting models is similar with or without an outlier. The influence of structurally diverse compounds is only minor, when compared to the changes in performance for the static approaches.

We repeated all calculations with design sets of different size (66% and 75% of compounds) for all datasets and found no significant difference to the results presented in this study.

## 5. Conclusion

The results of our study show that stepwise approaches, which take the correlation to the target property into consideration, significantly improved the quality of experimental design in terms of QSAR modeling. This observation is in agreement with the results of our recent study.[37] We recommend, whenever this is feasible, to design experiments in a stepwise manner. Especially in the case of high cost experiments, e.g. measuring aquatic bio-concentration factor,[38] that allow only a limited number of tests, the stepwise approaches can significantly decrease the financial effort to produce models of the same predictive quality. These models can be used to predict the molecules without measurements thus decreasing costs and time.

The PLS-Optimal approach is an appropriate choice for compounds and endpoints, where a linear correlation between the target property and the descriptor space is expected.[18] For other kinds of dependencies, DescRep shows a fast convergence in error, a reliable performance with a low standard deviation, and a high robustness against structural outliers. With respect to the structural outlier, it was dramatic to see how the majority of selection procedures were strongly affected with the inclusion of only one compound, which was not representative of the analyzed set. This resulted in higher variability of models developed with such sets.

Compared to the static approaches, the selection within stepwise approaches is not so focused on certain compounds, but on a harmonious context within the selection. Thus small variations in the dataset, as they were introduced by the random splits into design and validation set, get buffered in an efficient way.

The analyzed step-wise approaches, DescRep and PLS-Optimal design, explore different ideas for selection of compounds based on similarity and dissimilarity measures. Both methods produced comparable results. Thus, we can conclude that the major contribution to their performance was not the selection method, but the accounting for the resulting property, i.e. informational basis on which the selection was performed. Similar observations were done for QSAR modeling, where the underlying data, but not the chosen machine learning method or descriptors determined the accuracy of models.[39, 40, 41]

## 6. Software used

PLS models to evaluate the performance of the analyzed approaches were calculated with WEKA.[42]

## 7. Implementation and accessibility of data

The datasets used in this article are provided as excel tables in Supporting Information-1. To support the validity of the graphical representations provided in this article, the validation statistics are provided as Supporting Information-2. The datasets used in this article and the developed models are available at: http://ochem.eu/

## Supplementary Material

Robustness in experimental design: A study on the reliability of selection approaches

Robustness in experimental design: A study on the reliability of selection approaches
